# Physical activity, physical mobility, and mental health among persons 70 years or older: results from a large population-based study in Sweden

**DOI:** 10.1186/s13690-025-01718-w

**Published:** 2025-09-08

**Authors:** Malin Asp, Bo Simonsson, Anu Molarius

**Affiliations:** 1 Public Health Department, Region Värmland, Karlstad, Sweden; 2 Centre for Clinical Research, Region Västmanland, Västerås, Sweden; 3 Centre for Clinical Research, Region Värmland, Karlstad, 651 85 Sweden; 4 Department of Public Health Sciences, Karlstad University, Karlstad, Sweden

**Keywords:** Physical activity, Physical mobility, Mental health, Population studies

## Abstract

**Background:**

Physical inactivity, impaired physical mobility and poor mental health are common in the older population and increasing as the population ages. We examined the relationships between physical activity, physical mobility, and mental health in the general population of older adults.

**Methods:**

The study is based on 12 959 men and women aged 70 years or older answering a survey questionnaire sent to a random population sample in Mid-Sweden in 2022 (response rate 66%). The associations between physical activity (at least 150 min/week), physical mobility (no difficulties e.g. in walking short distances or climbing stairs) and mental health were investigated with multiple logistic regression analyses, adjusting for gender, age group and educational level. Mental health was measured with symptoms of anxiety or worry, and self-reported diagnosed depression.

**Results:**

In total, 51% were physically inactive and 31% had impaired physical mobility. Symptoms of anxiety or worry (33%) were more common among older people who were physically inactive than among those who were physically active (24%). The prevalence of symptoms of anxiety or worry was also higher among those with impaired physical mobility (40%) than among those with physical mobility (23%). Physical inactivity and impaired physical mobility were also associated with self-reported depression. When stratified by physical mobility, physical inactivity was significantly associated with symptoms of anxiety or worry (OR: 1.20, 95% CI: 1.08–1.34) and self-reported depression (OR: 1.66, 95% CI: 1.27–2.17) among those with physical mobility, whereas no association was found among those with impaired physical mobility.

**Conclusion:**

The results suggest that physical activity is positively associated with mental health among older people with physical mobility. However, about one in three in the older population has impaired physical mobility with poorer mental health irrespective of physical activity. These results are important for promoting mental health in an aging population.

**Supplementary Information:**

The online version contains supplementary material available at 10.1186/s13690-025-01718-w.

**Table:** 

**Text box 1. Contributions to the literature**
• The results of this large population-based study in Sweden imply that physical activity at least 150 min per week is positively associated with mental health among older people with physical mobility.
• The study also shows that about one in three persons 70 years or older has impaired physical mobility and they have poorer mental health irrespective of physical activity.
• The results suggest that it is important to invest in physical activity and physical mobility to promote mental health in an aging population.

## Background

Sweden has, as well as other European countries, an ageing population. Individuals in older ages are more likely than younger ones to have chronic diseases and functional impairment [1], which can increase the risk for depressive symptoms [2]. This leads to increased costs for health care and social systems associated with ageing [3].

Physical activity among older persons is important for preventing diseases, maintaining independence and quality of life [4]. Even moderate intensity of physical activity, like walking, helps to maintain mobility [5]. Physical activity is usually defined as a behaviour that involves bodily movements resulting in energy expenditure and includes exercise, sports, and physical activities performed as part of daily living, occupation, leisure, or active transportation [6, 7]. Exercise is a subcategory of physical activity that is planned, structured, and repetitive and that has as an objective for improvement or maintenance of physical fitness [7].

The prevalence of physical activity declines with age and is lowest among persons over 80 years [4, 8, 9]. This could be due to, among other things, the fact that physical mobility deteriorates with age. Impaired physical mobility usually implies decreased daily life activities such as walking short distances, climbing stairs, and showering [10, 11]. Physical mobility is the ability of a person to move independently in his/her environment, including walking, standing up from a chair, sitting down, and moving around. It is described as one of the six functional domains of disability in addition to seeing, hearing, cognition, self-care and communication [12].

Depression is the most common psychiatric disease worldwide [13]. The number of older adults with a lifetime history of depression will increase significantly over the next decades when the population is getting older [14]. Lower levels of physical activity are associated with poor mental health, both among younger [15] and older [16] adults. It seems that exercise in leisure time is more related to mental health than physical activity in other situations, for example household chores [17]. It is also important to be aware that older persons struggling with depression may refrain from leisure-related exercise [18]. In general, adults who exercise have fewer days of poor mental health in the past month than individuals who do not exercise, and that applies to all types of physical activities [15].

Previous studies have also shown that impaired mobility is an important risk factor for depression in older adults [19, 20]. In addition, depression can affect the earlier stages of impaired mobility [21]. However, previous studies have also reported contradicting findings what comes to the association between physical activity and depression or anxiety [22–24].

Possible causal pathways between physical activity, physical mobility and mental health include: (a) physical activity and physical mobility are independently associated with mental health; (b) physical mobility is a mediating (explanatory) factor between physical activity and mental health; (c) physical mobility modifies the association between physical activity and mental health and (d) mental health affects physical activity and/or physical mobility.

The findings of previous studies suggest that physical activity, physical mobility, and poor mental health are associated with each other. Since physical inactivity and impaired physical mobility are common in the older population, there is a need to investigate the associations between physical activity, physical mobility, and mental health simultaneously in this age group. This is important in order to acquire further knowledge about risk factors for mental health problems among older adults and for designing preventive activities to promote healthy aging and to decrease the individual and societal consequences of poor mental health in an aging population.

### Aim

The aim of this study was to examine the relationships between physical activity, physical mobility, and mental health among men and women 70 years or older in the general population.

## Methods

This study used data from a postal survey questionnaire (Life & Health Study) conducted in February-May 2022 [25]. The aim of the survey was to monitor health and its determinants in the general population. The questionnaire was sent to a random population sample of 78 000 persons aged 18 years or older in Mid-Sweden. Of them 19 992 were 70 years or older. In this age group, 13 123 persons (66%) responded. Of them 12 959 persons with non-missing data were included in the present study. The questionnaire comprised questions about lifestyle factors, living conditions and health. The sampling frame was the population register at Statistics of Sweden, the statistical administrative authority in Sweden, covering all inhabitants of the study area. The area investigated covers five counties (Sörmland, Uppsala, Värmland, Västmanland and Örebro), including 55 municipalities, comprising over one million inhabitants in the central part of Sweden. Data collection was discontinued after two postal reminders. The final reminder contained a shortened questionnaire with 15 questions with an attempt to increase the response rate.

### Physical activity

To assess physical activity, two questions were used. The first question was ‘How much time do you spend in a normal week on physical training that leaves you out of breath – for example running, fitness training, or boll sports?’. The response options were ‘0 minutes/no time’, ‘Less than 30 minutes’, ’30–59 minutes (0.5-1 hour)’, ’60–89 min (1–1.5 h)’, ‘90–119 min (1.5–2 h)’ and ‘2 h or more’. The second question was: ‘How much time do you spend in a normal week on daily activities, for example walking, cycling, or gardening? Count all time together’. The answer options were ‘0 min/no time’, ‘Less than 30 min’, ’30–59 min (0.5–1 h)’, ’60–89 min (1–1.5 h)’, ’90–149 min (1.5–2.5 h)’, ‘150–299 min (2.5–5 h) and ‘5 h or more’. An explanation text was given before the two questions: ’If your activities vary during the year, try to take some kind of average. Question (a) deals with regular exercise and training activities that leave you out of breath and sweaty, while (b) deals with moderately strenuous physical activity that leaves you somewhat short of breath, for example brisk walking, gardening, cycling or swimming.’ The questions are used in the national public health survey in Sweden to assess whether the respondent reaches 150 activity minutes of moderate-to-vigorous physical activity per week [26, 27] as recommended by the WHO [28]. The distributions of the two questions on physical activity are presented in Table S1. The number of activity minutes is calculated by taking the middle value in the range of the specified response. The minutes spent in physical training are doubled when the sum of the activity minutes from the two questions is calculated [26].

### Physical mobility

To assess physical mobility the questions used were ‘Can you run a short distance (about 100 m)?’. The response options were ‘Yes’ and ‘No’. If the answer was ‘no’, the next question was ‘Are you limited by your health in any of the following activities?’. The questions were ‘Can you walk up steps without difficulty? e.g. get on a bus or train’, ‘Can you take a short walk (about 5 minutes) at a reasonably fast pace?’ and ‘Do you need any aids or the help of another person to move around outdoors?’. The response options were ‘Yes’ and ‘No’ to each question. The participant was coded to have physical mobility if he/she answered yes to the first question or no to the first question but yes to all the three following questions about mobility and otherwise as having impaired physical mobility. These questions are identical to those used to assess physical mobility in the national public health survey in Sweden [26].

### Mental health

To assess mental health two questions were used. The first question was ‘Do you have any of the following diagnosed illnesses?’ with examples of diseases with one of them being depression. The response options were: ’Yes’ and ’No’. The second question about mental health was ‘Do you have any of the following discomforts or symptoms?’. One of the discomforts or symptoms was anxiety or worry, with the response options ‘No’, ’Yes, minor discomfort’ and ’Yes, severe discomfort’. To be considered to have symptoms of anxiety or worry it was required that the respondent answered option two or three to the question. The question on symptoms of anxiety or worry was identical to the question used in the national public health survey in Sweden [26].

### Covariates

Three covariates were adjusted for in this study: gender, age group and educational level. These variables were based on register data from Statistics Sweden. Age was grouped into 5-year intervals. Educational levels were divided into three groups; low (elementary school), medium (upper secondary school), and high (at least 3 years of university education).

### Ethical considerations

The study followed the Swedish guidelines for studies in social sciences and humanities, in accord with the Declaration of Helsinki [29]. In the information letters, it was clearly indicated that the respondent had the opportunity to decline participation in the study. In that case, no reminders were sent. The participants gave their consent by answering the questionnaire. After applying the register data from Statistics Sweden, all identity information was removed before the material was handed over to the respective Region for further processing. The information provided is protected according to the Public and Privacy Act 2009:400, Chap. 24, Sect. 8. The survey was approved by the Swedish Ethical Review Authority (DNR 2021-05814-01).

### Statistical analysis

The associations between physical activity, physical mobility, and mental health were analysed using chi squared test and multiple regression analysis. *P*-values < 0.05 were considered as statistically significant. In the multiple regression analyses, the outcome for mental health was symptoms of anxiety or worry, and self-reported diagnosed depression, respectively. The associations were adjusted for potential confounding factors gender, age group and educational level. An interaction term between physical activity and physical mobility was tested and statistically significant (*p* = 0.03, not shown) for depression but not for symptoms of anxiety or worry. Additionally, the analyses between physical activity and mental health were stratified by physical mobility. Results are reported as odds ratios (OR) and 95% confidence intervals (95% CI). SPSS, version 26, was used for all analyses.

## Results

Sociodemographic characteristics of the 12 959 persons included in the study are shown in Table 1. In total, 51% were physically inactive and 31% had impaired physical mobility. The table also shows the proportion of individuals with symptoms of anxiety or worry, and self-reported depression in relation to mobility, physical activity, and covariates gender, age group, and educational level. More women than men experienced anxiety or worry (36% vs. 22%). The prevalence of anxiety or worry increased with age and was lower among those with high educational level (25%) compared with those with medium and low educational level (29% and 31%, respectively). Older people who were physically inactive experienced more often anxiety or worry (33%) compared to older persons who were physically active (24%). Those with impaired physical mobility were more likely to experience anxiety or worry (40%) than persons with physical mobility (23%).

**Table 1:** Background characteristics of the study population and the prevalence of symptoms of anxiety or worry, and self-reported depression, in total and by independent variables (physical activity and physical mobility) and covariates (gender, age group, and educational level)

	*n* (%)	No anxiety or worry *n* (%)	Anxiety or worry *n* (%)	*p*-value	*n* (%)	No self-reported depression *n* (%)	Self-reported depression *n* (%)	*p*-value
Total	12 959 (100)	9 236 (71)	3 723 (29)		12 326 (100)	11 770 (95)	556 (5)	
Independent variable								
Physical activity				< 0.001				< 0.001
Physically active at least 150 min/week	6 257 (49)	4 770 (76)	1 487 (24)		6 031 (50)	5 848 (97)	183 (3)	
Physically inactive	6 439 (51)	4 300 (67)	2 139 (33)		6 067 (50)	5 709 (94)	358 (6)	
Physical mobility				< 0.001				< 0.001
Physical mobility	8 194 (69)	6 318 (77)	1 876 (23)		8 132 (70)	7 896 (97)	236 (3)	
Impaired mobility	3 616 (31)	2 181 (60)	1 435 (40)		3 550 (30)	3 275 (92)	275 (8)	
Covariates								
Gender				< 0.001				< 0.001
Women	6 385 (49)	4 087 (64)	2 298 (36)		6 077 (49)	5 718 (94)	359 (6)	
Men	6 574 (51)	5 149 (78)	1 425 (22)		6 249 (51)	6 052 (97)	197 (3)	
Age in years				< 0.001				0.789
70–74	4 215 (33)	3 124 (74)	1 091 (26)		4 073 (33)	3 892 (96)	181 (4)	
75–79	3 778 (29)	2 744 (73)	1 034 (27)		3 647 (30)	3 484 (95)	163 (5)	
80–84	2 038 (16)	1 410 (69)	628 (31)		1 903 (15)	1 822 (96)	81 (4)	
85 +	2 928 (23)	1 958 (67)	970 (33)		2 703 (22)	2 572 (95)	131 (5)	
Educational level				< 0.001				0.022
Low	4 125 (32)	2 858 (69)	1 267 (31)		3 850 (31)	3 663 (95)	187 (5)	
Medium	5 390 (42)	3 806 (71)	1 584 (29)		5 153 (42)	4 905 (95)	248 (5)	
High	3 365 (26)	2 516 (75)	849 (25)		3 254 (27)	3 135 (96)	119 (4)	

More women than men indicated self-reported depression (6% vs. 3%) (Table 1). The proportion of self-reported depression was about the same among people with high educational level (4%) compared with those with medium and low educational level (5%). Persons who were physically inactive also reported more often self-reported depression (6%) compared to those who were physically active (3%). In addition, persons with impaired physical mobility were more likely to indicate self-reported depression (8%) than those with physical mobility (3%).

The unadjusted OR confirms that there was a statistically significant association between physical activity and symptoms of anxiety or worry (Table 2). When adjusted for physical mobility, gender, age group, and educational level, the odds for experiencing symptoms of anxiety or worry were 1.18 times higher among persons who were physically inactive compared to those who were physically active. Older people with impaired physical mobility had 1.89 times higher odds for experiencing symptoms of anxiety or worry than those with physical mobility, when adjusted for the covariates. In addition, the adjusted odds for self-reported depression were 1.38 times higher among physically inactive compared to physically active persons. Older people with impaired physical mobility had 2.66 times higher odds for reporting self-reported depression than older people with physical mobility, when adjusted for the covariates.

**Table 2:** Unadjusted and adjusted odds ratios (OR) with 95% confidence intervals (CI) for symptoms of anxiety or worry and self-reported depression

	Anxiety or worry		Self-reported depression	
	**Unadjusted**	Adjusted*	**Unadjusted**	Adjusted*
N	12 696	11 523	12 098	11 424
	OR (95% CI)	OR (95% CI)	OR (95% CI)	OR (95% CI)
Physical activity				
Physically active at least 150 min/week (reference)	1.00	1.00	1.00	1.00
Physically inactive	1.60 (1.48–1.73)	1.18 (1.08–1.29)	2.00 (1.67–2.40)	1.38 (1.12–1.70)
Physical mobility**				
Physical mobility (reference)	1.00	1.00	1.00	1.00
Impaired physical mobility	2.22 (2.04–2.41)	1.89 (1.71–2.08)	2.81 (2.35–3.36)	2.66 (2.16–3.27)
Covariates				
Gender				
Men (reference)		1.00		1.00
Women		2.01 (1.85–2.19)		1.71 (1.41–2.06)
Age in years				
70–74 (reference)		1.00		1.00
75–79		1.03 (0.93–1.15)		0.86 (0.68–1.08)
80–84		1.04 (0.91–1.19)		0.72 (0.54–0.97)
85+		1.06 (0.93–1.20)		0.63 (0.48–0.82)
Educational level				
Low		1.19 (1.06–1.33)		1.23 (0.95–1.58)
Medium		1.17 (1.05–1.30)		1.22 (0.96–1.55)
High (reference)		1.00		1.00

* Adjusted for all the variables listed in the table

** *N* = 11 810 for anxiety and worry (unadjusted), *N* = 11 682 for self-reported depression (unadjusted)

**Fig. 1:**
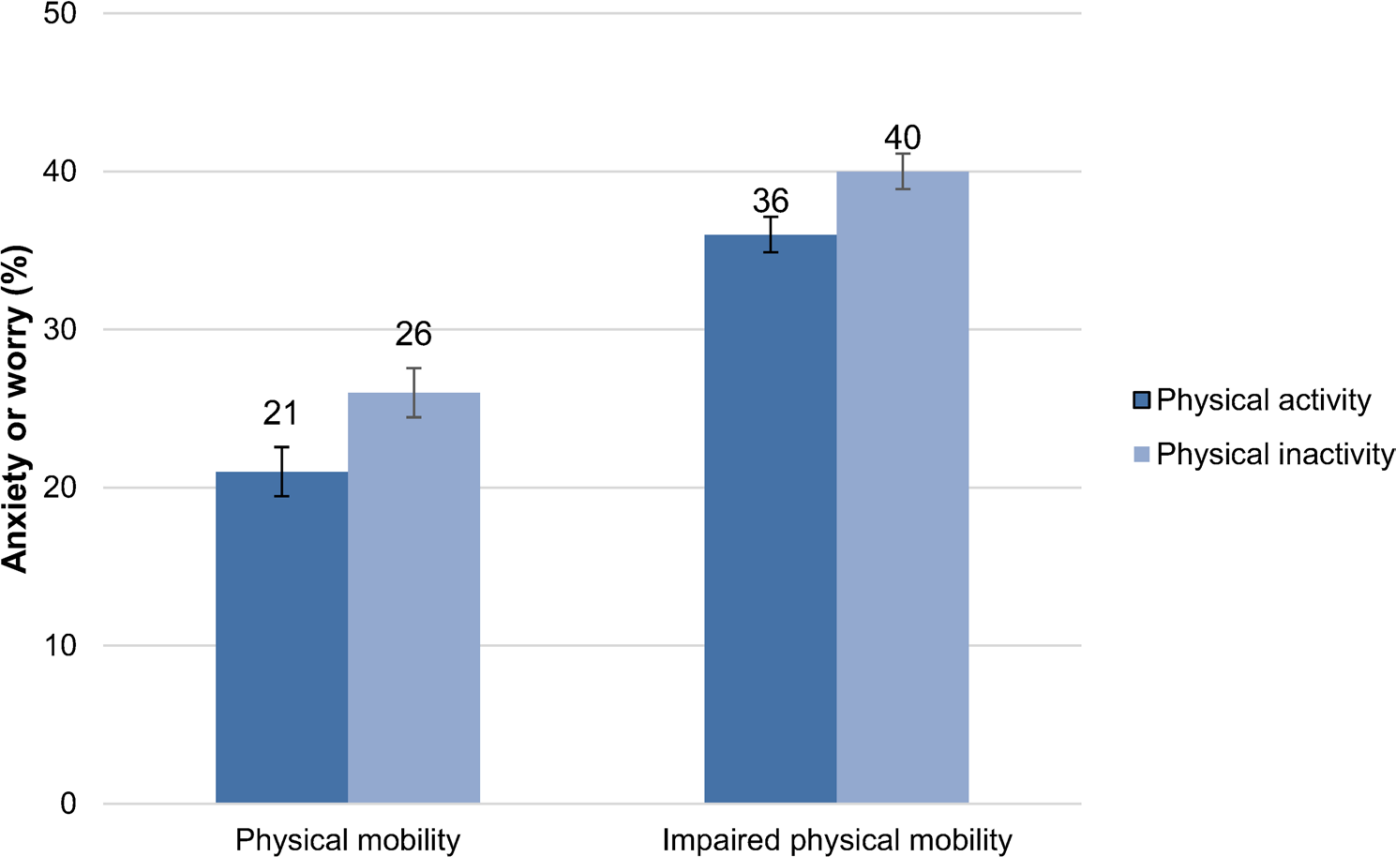
Prevalence of symptoms of anxiety or worry among physically active and inactive elderly stratified by physical mobility

Next, we analysed the association between physical activity and anxiety or worry stratified by physical mobility. In general, the prevalence of symptoms of anxiety or worry was higher among those with impaired physical mobility than among those with physical mobility (Fig. 1). Among those with impaired mobility, there were small differences between those who were physically active and inactive in the prevalence of anxiety or worry. However, among those with physical mobility, the prevalence was lower among physically active persons than inactive persons (*p < 0.05*).

**Fig. 2:**
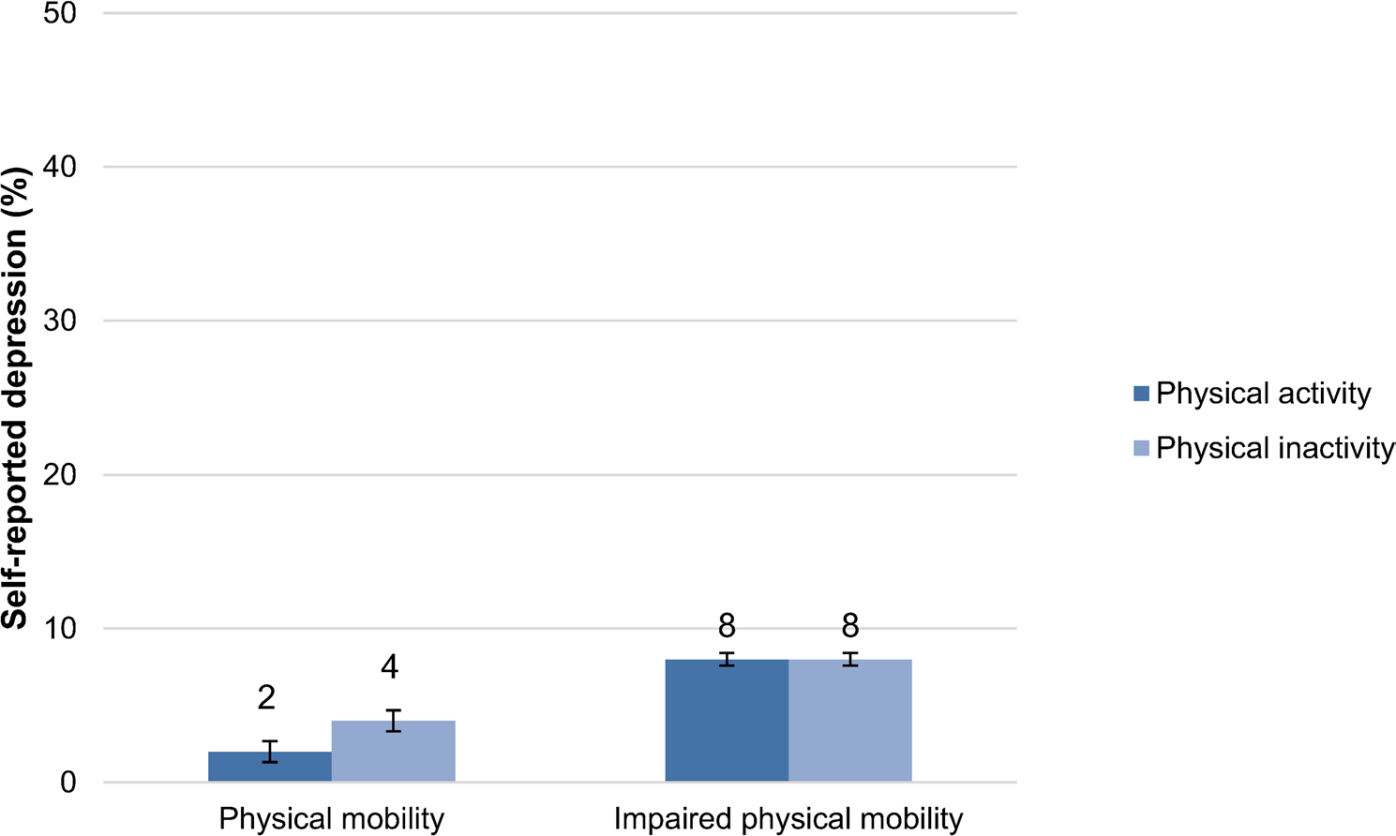
Prevalence of self-reported depression among physically active and inactive elderly stratified by physical mobility

Figure 2 shows that older people with impaired physical mobility had also more self-reported depression than older people who were physically mobile. Among those with physical mobility, self-reported depression was more common among physically inactive than physically active individuals (Fig. 2). Among individuals with impaired mobility, the prevalence of self-reported depression was equally high among physically active as among physically inactive individuals.

When stratified by physical mobility, the results of the logistic regression adjusted for gender, age group, and educational level confirm that physical inactivity was statistically significantly associated with symptoms of anxiety or worry among those with physical mobility (OR: 1.20, 95% CI: 1.08–1.34) but not among those with impaired physical mobility (OR: 1.13 95% CI: 0.95–1.34) (Table 3). In addition, adjusted odds ratios for self-reported depression show that physical inactivity (OR: 1.66, 95% CI: 1.27–2.17) had a strong association with depression among older people with physical mobility. However, there was no association (OR: 1.06, 95% CI: 0.78–1.45) between physical inactivity and depression among those with impaired physical mobility.

**Table 3:** Adjusted odds ratios (OR) with 95% confidence intervals (CI) for symptoms of anxiety or worry, and self-reported depression, stratified by physical mobility*

	Anxiety or worry		Self-reported depression	
	**Physical mobility**	**Impaired physical mobility**	**Physical mobility**	**Impaired physical mobility**
N	8 048	3 475	8 000	3 424
	OR (95% CI)	OR (95% CI)	OR (95% CI)	OR (95% CI)
Physical activity				
Physically active at least 150 min/week (reference)	1.00	1.00	1.00	1.00
Physically inactive	1.20 (1.08–1.34)	1.13 (0.95–1.34)	1.66 (1.27–2.17)	1.06 (0.78–1.45)
Covariates				
Gender				
Men (reference)	1.00	1.00	1.00	1.00
Women	2.20 (1.97–2.45)	1.72 (1.49–1.98)	1.53 (1.17–2.00)	1.91 (1.46–2.51)
Age in years				
70–74 (reference)	1.00	1.00	1.00	1.00
75–79	1.07 (0.95–1.22)	0.93 (0.75–1.16)	0.88 (0.65–1.19)	0.83 (0.58–1.19)
80–84	1.05 (0.89–1.23)	1.01 (0.81–1.28)	0.79 (0.52–1.20)	0.66 (0.44–1.00)
85+	1.08 (0.90–1.29)	1.00 (0.82–1.22)	0.63 (0.39–1.02)	0.62 (0.44–0.87)
Educational level				
Low	1.15 (1.00–1.33)	1.25 (1.03–1.51)	1.15 (0.80–1.66)	1.28 (0.89–1.83)
Medium	1.19 (1.05–1.36)	1.14 (0.94–1.37)	1.25 (0.90–1.73)	1.18 (0.83–1.68)
High (reference)	1.00	1.00	1.00	1.00

* Adjusted for all the variables listed in the table

## Discussion

In the present population-based study, 51% of the subjects 70 years or older were physically inactive and 31% had impaired physical mobility. Older people who were physically inactive experienced more often symptoms of anxiety or worry and self-reported depression compared to older persons who were physically active. The prevalences of symptoms of anxiety or worry and depression were also significantly higher among those with impaired physical mobility than among persons with physical mobility. However, when stratified by physical mobility, physical inactivity was significantly associated with symptoms of anxiety or worry and self-reported depression only among those with physical mobility, whereas no association was found among those with impaired physical mobility.

In line with the findings of our study, a number of previous studies among older adults have reported an association between physical inactivity and depression [16, 18, 30–32]. Moreover, a bidirectional association between physical inactivity and symptoms of depression and anxiety has been found which was confirmed by Azevedo Da Silva et al. [22] in the longitudinal Whitehall Study. However, some studies have found an inverse association between physical activity and anxiety but not between physical inactivity and depression [23, 24]. In the current study, physical inactivity was significantly associated with both symptoms of anxiety/worry and self-reported depression. The prevalence of symptoms of anxiety or worry (29%) was higher than the prevalence of diagnosed depression (5%) and the odds ratio for physical inactivity was highest (1.66) for depression among those with physical mobility.

There is also convincing evidence that depression increases the subsequent risk for physical disability and, in turn, physical disability results in increased depressive symptoms [19, 20, 33, 34]. In addition, an association between physical disability and anxiety has been demonstrated among older people [19]. While our study included only one type of physical disability i.e. physical mobility, a strong relationship between physical mobility and both symptoms of anxiety/worry and self-reported depression among older adults was found in the current study.

Since physical activity is associated with gender and age [18] in older adults and older age and low education are associated with impaired mobility [35] we adjusted for gender, age group and educational level in the logistic regression analyses. Previous studies have adjusted for similar factors. Loprinzi [31] found among older US adults that even after controlling for gender, age, ethnicity, marital status, education, body mass index, comorbidity index and physical function, physical activity was inversely associated with depression. Weyerer et al. [34], in turn, found gender and older age but not educational level to be associated with increased incidence of depressive disorders in their study of predictors of depression.

Physical activity, physical mobility and depression are known to be associated with each other. For example, Wassink-Vossen et al. [16] found that those aged 60 and over with depression are less physically active in comparison with their non-depressed counterparts and that functional limitations are a major explanatory factor for this difference in physical activity. It has been suggested that difficulty in walking leads to reduced physical activity and limited living space, which in turn leads to a loss of social interaction and support, which in turn leads to depressive symptoms [36]. In addition, a decrease in physical activity and living space limitations can lead to a decline in physical function [36].

To our best knowledge, few previous studies have investigated the association between physical activity and mental health stratified by physical mobility in a general population of older adults. A relatively small Finnish population-based longitudinal study of older adults (*N* = 384) found a strong association between impaired mobility and depressive symptoms but no significant interaction between physical activity and mobility in predicting the development of depressive symptoms [37]. A large cross-sectional study among adults aged 18 years or older in low and middle-income countries (*N* = 178 867) found that individuals with depression engage lower levels of physical activity [38]. A large part of this association was explained by mobility limitations i.e. mobility limitations were a major mediating factor between depression and physical activity [38].

In summary, our study showed that physical inactivity is significantly associated with symptoms of anxiety/worry and self-reported depression among older people with physical mobility. However, as many as one third of the older population has impaired physical mobility. They have poorer mental health than those with physical mobility irrespective of physical activity.

### Strengths and limitations

This study has several strengths. An important strength is the high response rate of 66%. It is common that the response rate is around 50% in population surveys [39], or even lower among very old people [40]. A high response rate increases the representativeness of the studied population and decreases the risk of bias of estimates. The response rate was relatively similar among men and women. However, the response rate was lower in the age group 85 years or older (53%) than in the age group 70–84 years (71%). In addition, a lower response rate among those with low educational level compared to those with high educational level was detected. The study was population-based and included a large sample (*n* = 12 959). In addition, a large age range of older persons over 70 years, including those 85 years or older, was studied. The age group 70 years or older was selected since, in contrast to those 65–69 years, very few persons in this age group are working and therefore physical activity at work is not affecting the results. We used two different types of measures of mental health, one based on symptoms (anxiety/worry), and one based on diagnosed illness (depression), to confirm the findings.

This study has also some limitations. These include the cross-sectional design, which does not allow determining cause and effect. Even though the results of the current study suggest that physical mobility may be an effect modifier between physical activity and mental health, future research should carry out longitudinal studies to investigate the different causal pathways between physical activity, physical mobility and mental health in older adults. The current study is limited to the national context in Sweden and five counties. Another limitation regards the dichotomization of the measures which might reduce the specificity of the data. Physical activity was self-reported which may introduce bias [6]. However, using direct measures of physical activity such accelerometers is often not feasible in large population surveys. In addition, the questions on physical activity did not specifically mention transportation or household tasks (except gardening) and muscle strengthening exercise, and did not specify what is meant with “a normal week”. However, the questions are regarded as adequate for measuring physical activity at least 150 min/week [26, 27].

There are also some missing data for the variables included in the study, varying between 1% and 11%. Higher levels of missing data for physical mobility and self-reported depression are partly due to these variables not being included in the short version of the survey. In addition, the questions about physical mobility and physical activity contained several questions which may have increased the proportion of missing data. Missing values and non-response are also both related to mental health [41] which could lead to underestimating the prevalence of depression. Anxiety and worry may be more difficult to study in older adults as they are less accurate in identifying anxiety symptoms and tend to minimize symptoms and to attribute symptoms to physical disability [42]. Further, this study used a self-report of diagnosed depression. However, a previous study has shown that self-reported clinician diagnosed depression during the last 12 months is associated with at least one DSM-IV mental disorder [43] and another study found that the validity of self-reported clinician diagnosed depression is adequate to assess depression status [44]. Since the survey was conducted among the adult general population (18 years or older) it was not possible to include measures specifically designed for an older population, such as Geriatric Depression Scale [32, 34] or Geriatric Anxiety Inventory [45].

Yet another limitation is that we did not adjust for health behaviours (such as smoking or alcohol consumption), body mass index, comorbidity, cognitive limitations or urban/rural residence which may have been additional potential confounders. Previous studies have, however, found an association between physical activity or physical mobility and mental health even when adjusting for health behaviours [33, 34, 36], body mass index [31–33], comorbidity [31, 34] or cognitive limitations [33, 34]. In addition, we performed a complementary analysis adjusting also for daily smoking, risk consumption of alcohol and obesity (body mass index ≥ 30 kg/m^2^). Risk consumption of alcohol and obesity were associated with symptoms of anxiety or worry and daily smoking with diagnosed depression (*p* < 0.05, not shown), but the odds ratios for physical activity and physical mobility remained unchanged.

## Conclusions

Physical activity (at least 150 min/week) was positively associated with mental health among older people with physical mobility. However, about one in three in the population over 70 years has impaired physical mobility with poorer mental health irrespective of physical activity. Results in the present study emphasize that, regardless of the different backgrounds of the participants, it is important to promote physical activity among persons with physical mobility and to invest in physical mobility to promote mental health in an aging population.

## Supplementary Information

Below is the link to the electronic supplementary material.

Supplementary Material 1

## Data Availability

The data materials used in the current study are not publicly available due to confidentiality and regulations under the Swedish law (Public and Privacy Act 2009:400, Chapter 24, Sect. 8), but descriptive data in table form are available from the corresponding author on reasonable request.

## Abbreviations

CI: Confidence interval
OR: Odds ratio
SPSS: Statistical Package for the Social Sciences
WHO: World Health Organization
