# Supplementary material for: Physical activity, physical mobility, and mental health among persons 70 years or older: results from a large population-based study in Sweden
Source: Arch Public Health. 2025 Sep 8;83:225. doi: 10.1186/s13690-025-01718-w (PMC12418685; doi:10.1186/s13690-025-01718-w)
Supplement: Supplementary file 1 — Supplementary Material 1 [file 13690_2025_1718_MOESM1_ESM.docx]

Table S1. Distribution of the two questions on physical activity among subjects aged 70 years or older.

|  |  | **N** | **%** |
| --- | --- | --- | --- |
| **Physical training per week** | No time | 4 919 | 39.3 |
|  | < 30 min | 3 126 | 25.0 |
|  | 30-59 min | 1 932 | 15.4 |
|  | 60-89 min | 909 | 7.3 |
|  | 90-119 min | 534 | 4.3 |
|  | 120+ min | 1 089 | 8.7 |
|  |  |  |  |
| **Daily activities e.g. walking, cycling or gardening per week** | No time | 1 100 | 8.6 |
|  | < 30 min | 1 633 | 12.8 |
|  | 30-59 min | 2 369 | 18.6 |
|  | 60-89 min | 1 640 | 12.9 |
|  | 90-149 min | 1 770 | 13.9 |
|  | 150-299 min | 2 060 | 16.2 |
|  | 300+ min | 2 147 | 16.9 |
